# Molecular Profiling of Inflammatory and Myofibroblast Cancer-Associated Fibroblast Subtypes Derived from Human Pancreatic Stellate Cells Using Machine Learning-Based Label-Free Raman Spectroscopy

**DOI:** 10.34133/bmr.0292

**Published:** 2025-12-09

**Authors:** Minju Cho, Eun-Young Koh, Yeounhee Kim, Seong-Jin Kim, Chan-Gi Pack, Eunsung Jun, Jun Ki Kim

**Affiliations:** ^1^ Department of Convergence Medicine, Brain Korea 21 Project, University of Ulsan, College of Medicine, Asan Medical Center, Seoul, Republic of Korea.; ^2^ Department of Biomedical Engineering, University of Ulsan College of Medicine, Asan Medical Center, Seoul, Republic of Korea.; ^3^ Division of Hepato-Biliary and Pancreatic Surgery, Department of Surgery, University of Ulsan College of Medicine, Asan Medical Center, Seoul, Republic of Korea.; ^4^ Biomedical Engineering Research Center, Asan Medical Center, Seoul, Republic of Korea.

## Abstract

Cancer-associated fibroblasts (CAFs), one of the most substantial constituents of the pancreatic tumor microenvironment, exhibit far greater heterogeneity and phenotypic plasticity than it was previously recognized. Accordingly, distinguishing between CAF subpopulations and their functional roles in pancreatic tumorigenesis has become increasingly important. Additionally, as the importance of the therapeutic approach increases, interests in technologies capable of efficiently differentiating between normal fibroblast subpopulations and pathologic CAFs also grow. Label-free imaging and analytical technologies that do not require fluorescent labeling or other preprocessing steps offer a promising alternative to conventional invasive cell analysis. Here, we employed Raman spectroscopy to chemically characterize human primary pancreas stellate cell (HPaSC), inflammatory CAF (iCAF), and myofibroblastic CAF (myCAF) derived from HPaSC at the cellular level for molecular profiling. As a result, we successfully compared the distinctive biological and chemical properties of each fibroblastic subtype. These Raman spectrum findings were validated by transcriptomic and lipidomic analysis. Our molecular profiling demonstrates that CAF subpopulations can be quantitatively distinguished based on their intrinsic chemical signatures, offering valuable insights into identifying and characterizing CAFs without relying on fluorescence or specific biomarkers. These multivariate spectral analyses enable subtype classification in 95% accuracy combined with partial least squares discriminant analysis (PLS-DA). This result demonstrates that CAF subtypes can be quantitatively distinguished using their intrinsic molecular signature, which support potential in pancreatic cancer research and therapeutic development.

## Introduction

Pancreatic ductal adenocarcinoma (PDAC) accounts for >85% of pancreatic cancer and is associated with a poor prognosis, with a 5-year survival rate of only 10% [1]. Despite advancements in research and treatment, PDAC remains the second leading cause of cancer-related deaths. The disease progresses silently until symptoms manifest, often when metastasis to other organs has occurred, leading to delayed diagnosis. Consequently, early diagnosis for PDAC is challenging, and many patients are diagnosed at a stage where surgical intervention is not feasible and are subsequently treated with chemotherapy.

Therapeutic approaches for PDACs face considerable challenges, largely due to them being based on genetic and cellular complexities of cancer cell [2]. Studies have shown that PDAC tumors exhibit subtypes varying metastatic abilities, which may respond differently to specific treatments [3]. The tumor microenvironment (TME) of PDAC is characterized by desmoplastic and extensive immunosuppressive properties, both of which contribute to tumor progression and limit treatment efficacy. The TME is a dynamic environment surrounding the tumor cells, shaped by the molecular and cellular physical characteristics of the cancer; this microenvironment induces changes within tissues and plays a pivotal role in promoting cancer progression [4]. From the early stages of tumor growth, cancer cells engaged in dynamic and interactive relationships with the TME components, allowing them to survive, infiltrate locally, and metastasize. Therefore, TME has become a crucial focus in recent drug development studies.

TME comprises various cellular and noncellular components, including immune cells, endothelial cells, and CAF cytokines [5]. CAFs are one of the most abundant matrix cell types in TME, and they promote tumor progression by interacting with tumor and immune cells through the reconstruction of extracellular matrix (ECM) and secretion of various growth factor chemokines and cytokines [6]. CAF subtypes have been identified based on specific gene expression patterns and are distributed differently within the tumor and TME. There are 2 CAF subtypes, namely, α-SMA (α-smooth muscle actin) high interleukin-6 (IL-6) low myofibroblasts (myCAFs) and α-SMA low IL-6 high inflammatory CAFs (iCAFs) [7]. myCAFs can exert either tumor-suppressive or tumor-promoting effects depending on the TME and progression stage. In addition, myCAF secretes various ECM components, such as collagen, laminin, fibronectin, proteoglycan, and periostin, which promote tumor progression and metastasis. myCAFs also produce proteases, such as matrix metalloproteinase (MMP), and facilitate tumor cell infiltration [8]. Conversely, iCAFs secrete inflammatory cytokines and growth factors, which eventually create and sustain an inflammatory state within the TME environment, expressing CXCL12 or IL6 [8]. iCAFs promote tumors by inducing cancer cell proliferation, metastasis, and resistance to anticancer drugs. Identifying CAF phenotype is crucial for advancing research on anticancer treatment. Current CAF research often relies on techniques, such as fluorescence immunostaining, RNA sequencing, and immunoassay, which involve invasive procedures that require attaching specific labels to cells. Since various immunostaining performed through this process transforms the physical properties of the cell, the minimum invasive cell manipulation method should be considered [9]. Thus, we attempted to compare and analyze the properties of myCAFs and iCAFs using label-free Raman spectroscopy.

Raman spectroscopy is a noninvasive analytical technique that utilizes Raman scattering phenomenon, where laser interacts with the chemical bond vibration of the object, causing changes in frequency from the original photon energy [10,11]. This Raman scattering reflects the molecular composition of the object and can be used to analyze biomolecular properties, such as proteins, lipids, and nucleic acids. Here, we hypothesize that the intrinsic chemical signatures of CAF subtypes differ enough to be detected by Raman spectroscopy.

**Fig. 1.**
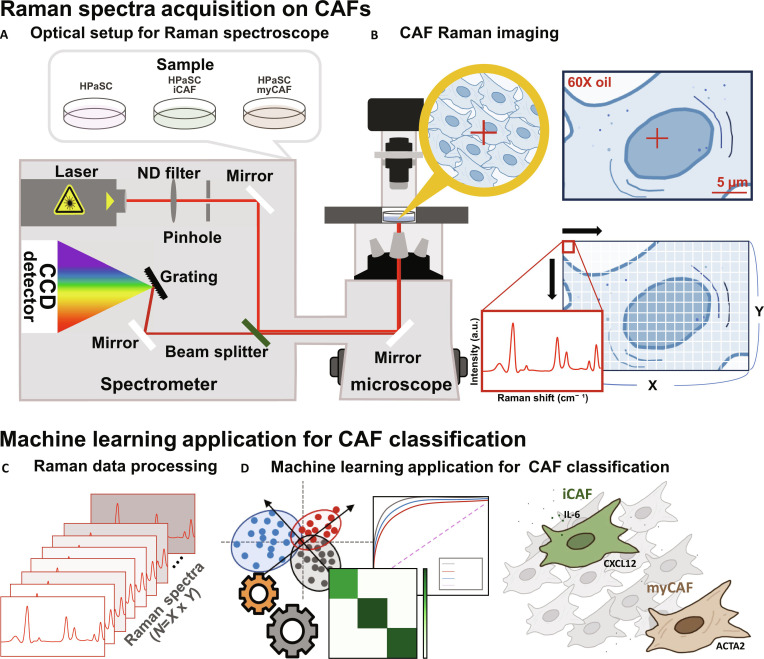
Schematic workflow of CAF subtype identification using Raman spectroscopy and artificial intelligence (AI)-based analysis. (A) Optical setup for Raman spectroscope. The optical setup includes a laser source, pinhole, grating, and charge-coupled device (CCD) detector within a spectrometer system coupled to a microscope. (B) CAF Raman imaging was performed as Raman mapping, which individual spectrum recorded at each point along the *x* and *y* pixels of mapping size. (C) Data preprocessing was performed on the acquired Raman spectra. (D) For CAF classification, machine learning was applied. The confusion matrix and ROC for evaluation of classification accuracy.

In this study, we employed Raman spectroscopy to analyze the characteristics of myCAFs and iCAFs. Raman signals were subjected to partial least squares discriminant analysis (PLS-DA) for cellular discrimination with the extraction of important molecular features. Accordingly, we have successfully distinguished human pancreatic stellate cell (HPaSC), myCAF, and iCAF induced by HPaSC (Fig. 1). HPaSCs, which are the predominant fibroblast in the pancreas, store lipid and express intermediate filament [12]. Since CAFs originate from HPaSC under the influence of tumor-related signals, such as cancer cell-derived chemokines, cytokines, and microRNAs [13], HPaSCs transform into myoblast-like structure CAFs, expressing their unique transcriptional characteristics in PDAC. To simulate the CAF transformation, we selected HPaSC to induce CAFs. To assess the accuracy of cell classification, we performed confusion matrix and receiver operating characteristic (ROC) analysis. While analyzing the spatial distribution and metabolic characteristics of CAFs in tumor tissues from patients with pancreatic cancer through spatial transcriptomics, the main component Raman peak obtained from PLS-DA was compared with the biological characteristic of CAFs. Through this analysis, the role of myCAFs and iCAFs in TME is more precisely identified and its functionality as a therapeutic target is evaluated.

Up to date, Raman spectroscopy has been applied to discriminate cancer cell type, but the discrimination and classification of CAF subtypes using Raman spectroscopy with combined machine learning have not been reported. We demonstrate that Raman spectroscopy combined with machine learning enables label-free, noninvasive, and rapid classification of iCAFs and myCAFs derived from HPaSCs. Raman-based analysis combined with machine learning to elucidate distinct CAF subtypes provides new insights into CAF heterogeneity and lays a foundation for improved therapeutic stratification in pancreatic cancer.

## Materials and Methods

### Analysis of spatial transcriptomics data

#### Human sample

Human PDAC tumor tissue was gained from the BioResource Center (BRC) of Asan Medical Center, Seoul, Republic of Korea. The Institutional Review Board (IRB) of Asan Medical Center (IRB approval number: 2021-0988) approved this study. Prior to the sample acquisition, informed consent was obtained from patients. Sample was confirmed as tumor tissues by pathological assessment conducted by 2 or more independent pathologists.

#### scRNA-seq and data preprocessing

Single-cell RNA sequencing (scRNA-seq) libraries were prepared using the Visium Spatial Gene Expression for FFPE kit (10× Genomics, CA, USA) following the manufacturer’s protocol. These RNA libraries were sequenced on the Illumina HiSeq XTEN platform (Illumina, San Diego, CA, USA). Initial quality control by Macrogen Inc. (Seoul, South Korea) was conducted on raw sequencing data. This process included the removal of low-quality reads, adapter trimming, and filtering of low-complexity sequences. Illumina base call files for all libraries were converted to FASTQs using bcl2fastq v2.20.0 (Illumina). Human sequencing reads were aligned to GRCh38-2020-A genome with Space Ranger count 2.0.0 (10x Genomics, CA, USA).

#### Data processing of spatial gene expression

Gene expression data were analyzed using Seurat. Read count matrices were normalized using the NormalizeData function. Spots were grouped based on expression profiles using the principal components analysis (PCA) approach. Clustering was performed using FindNeighbors and FindClusters (resolution = 0.3), resulting in 4 clusters, and differentially expressed genes (DEGs) were identified in each cluster. Uniform Manifold Approximation and Projection (UMAP) was applied for dimensionality reduction using the RunUMAP function. Two-dimensional (2D) UMAP plots were visualized using the DimPlot and FeaturePlot functions. Marker genes were identified using FindMarkers, and clusters were manually annotated based on known marker genes.

#### Identification of major cell types

The cancer-associated fibroblast (CAF) network was classified into iCAF and myCAF based on gene expression markers (iCAF signature genes: IL6, PDGFRA, CFD, PLA2G2A, HAS1, CXCL2, CCL2, CLU, EMP1, and LMNA; myCAF signature genes: TAGLN, ACTA2, MMP11, PDGFRB, HOPX, and POSTN). One ambiguous cluster was categorized as “other” [14]. DEGs between myCAF and iCAF were identified using FindMarkers (criteria: *P* ≤ 0.05, fold change > 2.5 for myCAF; *P* ≤ 0.05, fold change < −2.5 for iCAF). Gene Ontology (GO) and pathway enrichment analysis were conducted using Enrichr and gprofiler2, including GO Molecular Function (2023), GO Cellular Component (2023), GO Biological Process (2023), KEGG (2021) Human Pathways, Reactome (2022), The Kinase Library (2024), and WikiPathways (WP). Visualization and data statistical analyses were performed using the following R packages (v 4.4.0): devtools (v2.4.5), DoMultiBarHeatmap (v0.1.0), dplyr (v1.1.4), enrichR (v3.2), ggplot2 (v3.5.1), gprofiler2 (v0.2.3), Matrix (v1.7.0), Scillus (v0.5.0), scCustomize (v3.0.1), and Seurat (v5.0.3).

### Cell preparation of HPaSCs and induction of CAF subtype

HPaSCs were prepared for induction into activated phenotypes (myCAF or iCAF) to investigate their roles in tumor progression. HPaSCs were purchased from ScienCell (no. 3830, CA, USA) and cultured according to the manufacturer’s instructions. The cells were seeded in 60-mm dishes and treated with 20 ng/ml of human transforming growth factor-β1 (TGF-β1) (T7039-2UG, Merck, USA) for 4 d to induce the myCAF phenotype [15]. Similarly, 1 ng/ml of human IL-1α (200-LA-002, R&D Systems, USA) was administered for 4 d to induce the iCAF phenotype.

### Acquisition of Raman spectrum for CAFs

A Raman spectroscope (WEVE, Seong-nam, South Korea) with a 60× oil objective lens (LUCPLFLN40X, Olympus, NA = 0.6, WD = 2.7 to 4.0) was used for the measurements. The process utilized a 532-nm laser from 532-nm laser supply (CNI laser, Changchun, China) with the output power of 64.49 mW. The 2.9999-s exposure time was applied with the center value of 2,950 cm^−1^ using a 600-groove visual grating in addition to 80% ND filter application. HPaSC, HPaSC-induced iCAF, and HPaSC-induced myCAF were prepared in Raman dish under 4% paraformaldehyde (PFA) fixation. Total number of 8 cells for HPaSC, 10 cells for iCAF, and 12 cells for myCAF were imaged and analyzed. Through Raman mapping imaging, multiple spectra were collected from each cell by 1-μm intervals across both *X* pixel and *Y* pixels. Although the number of *X* and *Y* pixels varied depending on the cell size, at least 100 points of Raman spectrum from one sample were collected. Using the Raon-Vu program, background noise was removed after polynomial fitting and smoothing process. For Raman spectrum analysis, all samples were normalized at 1,040 cm^−1^, which is PFA signal [16].

### Machine learning algorithm on Raman spectrum data for cell classification

PLS-DA was conducted to extract variable importance in projection (VIP) using Python 3.11 (Python Software Foundation, Wilmington, DE, USA) and Visual Studio Code (v1.92, Microsoft, Redmond, WA, USA). We then constructed box plot for top VIP score to identify significant spectral feature that contributes to the classification between groups. We also evaluated accuracy, sensitivity, and specificity through confusion matrix and ROC based on the result of PLS-DA. We separated the whole Raman spectrum with 70% training set and 30% test set. Subsequently, the area under the curve (AUC) was derived. For the implementation of PLS-DA and visualization of graphs, multiple Python libraries were employed. The scikit-learn library was used to perform partial least squares regression (PLSRegression), as well as to split the dataset into training and test sets using train_test_split. The SciPy.Stats module was applied to conduct independent 2-sample *t* tests for statistical significance. Performance confusion matrix, AUC, and ROC curves were computed using functions from “sklearn.metrics” for classification performance. Additionally, matplotlib and seaborn libraries were utilized for visualization of box plots, Raman spectrum graph, VIP-based comparisons, and ROC curves.

### Lipidomic analysis

#### GC/MS

For free fatty acid, 1 million cells were lysed and mixed well with 1 ml of cold methanol and 50 μl of internal standard solution (0.1 mg/ml myristic acid-d14). Sample solutions were acidified with HCl to 25 mM final concentration and centrifuged. Supernatants were collected into fresh glass tubes, and 4 ml of iso-octane was added. The upper phase was collected after liquid–liquid extraction process and dried under vacuum. The dried sample was reacted with 200 μl of BCl3-MeOH, 12% w/w (Sigma-Aldrich) at 60 °C for 30 min. Subsequently, 100 μl of H_2_O and 100 μl of hexane were added sequentially, and the sample was mixed vigorously [17]. The upper phase was collected after resting the sample for 5 min. Then, 20 to 30 mg of anhydrous sodium sulfate were added and the supernatant was ready for gas chromatography/mass spectrometry (GC/MS) analysis using an Agilent 7890A gas chromatograph coupled with a 5975C mass selective detector (Agilent Technologies, Santa Clara, CA, USA). Commercially available fatty acid methyl esters (Sigma-Aldrich) were used to generate calibration curves without derivatization.

#### LC-MS/MS

Ceramide, sphingomyelin, fatty acid amides, and fatty acyl coenzyme As (CoAs) were analyzed using LC-MS/MS equipped with 1290 HPLC (Agilent, Waldbronn, Germany) and QTRAP 5500 (ABSciex, Toronto, Canada). Phospholipid profiling was analyzed using LC-MS/MS equipped with 1290 HPLC (Agilent, Waldbronn, Germany) and Triple Quadrupole 6500 (ABSciex, Toronto, Canada).

Cells were collected using 1.4 ml of cold methanol/H_2_O (80:20, v/v) after rapid and sequential washing with phosphate-buffered saline (PBS) and H_2_O. Subsequently, cells were lysed with vigorous vortex, and 50 μl of internal standard solutions [C18 ceramide-d7 for ceramides, 18:1 SM-d9 for sphingomyelins; 500 nM of arachidonoyl ethanol-amide-d4, oleoyl ethanol-amide-d4 for fatty acid amides, 5 μM malonyl-13C3 CoA for fatty acyl CoAs, 1 μM 18:0 D70-18:0 phosphatidylcholine (PC), 1 μM 16:0 D31-18:1 phosphatidylethanolamine (PE) for phospholipid profiling] was added [18]. Metabolites were collected from lower organic and upper aqueous phases using liquid–liquid extraction followed by adding chloroform and H_2_O. The aqueous and organic phase was dried using vacuum centrifuge and stored at −20 °C until liquid chromatography-MS/MS (LC-MS/MS) analysis.

The dried matter from the organic solutions was reconstituted with 50 μl of MeOH for lipids, and the aqueous solutions was reconstituted with 50 μl of 50% acetonitrile for fatty acyl CoAs and injected into the LC-MS/MS system. Metabolite and internal standards were purchased from Sigma-Aldrich and Avanti Polar Lipids, respectively. All solvents including water were purchased from J.T. Baker (Avantor, Radnor, PA, USA). The internal standards were purchased from Sigma-Aldrich or CDN Isotopes.

For ceramides and sphingomyelin, Zorbax Eclipse C18 (50 × 2.1 mm) was used with mobile phase A (10 mM ammonium acetate in MeOH/isopropanol/H_2_O, 900:50:50) and mobile phase B (10 mM ammonium acetate in MeOH/isopropanol/H_2_O, 940:50:10). The LC run was performed with the isocratic condition of 60% phase B. The flow rate was maintained at 400 μl/min for 20 min. For fatty acid amides, Pursuit5 C18, 150 × 2.1 mm was used with mobile phase A (0.1% formic acid in H_2_O) and mobile phase B (0.1% formic acid in MeOH). The LC run was performed with the isocratic condition of 90% B. Flow rate was maintained at 200 μl/min for 20 min.

For fatty acyl CoAs, Zorbax 300 Extend-C18 column (2.1 × 150 mm) was used. Mobile phase A was acetonitrile/H_2_O (10:90) with 15 mM ammonium hydroxide, and mobile phase B was acetonitrile with 15 mM ammonium hydroxide. The separation gradient was performed at the sequential process of holding at 0% B for 3 min, 0% to 50% B for 2 min, 50% to 80% B for 5 min, 80% to 0% B for 0.1 min, then 0% B for 4.9 min. LC flow was 200 μl/min. All column temperature was set at 25 °C for ceramides and sphingomyelin, fatty acid amides, and fatty acyl CoAs.

For phospholipid profiling, Zorbax Eclipse Plus C18 column (2.1 × 50 mm) was used with mobile phase A [10 mM ammonium acetate in MeOH/IPA/H_2_O (900:50:50, v/v/v)] and mobile phase B [10 mM ammonium acetate in MeOH/IPA/H_2_O (940:50:10, v/v/v)]. The separation gradient was performed at the sequential process of holding at 60% B for 10 min, 60% to 90% B for 0.1 min, 90% for 7.9 min, 90% to 60% B for 0.1 min, 60% B for 6.9 min. LC flow was 300 to 400 μl/min, and the column temperature was maintained at 23 °C. Multiple reactions monitoring (MRM) was performed in positive ion mode, and the extracted ion chromatogram (EIC) corresponding to the specific transition for each metabolite was used for quantitation. The AUC of each EIC was normalized to that of the internal standard. Generally, the calibration range for each analyte was 0.1 nM to 10 μM with *R*^2^ > 0.99. Quantitation result was normalized to the protein amount in a sample. Data analysis was performed using Analyst 1.7.1 software.

## Results

### Spatial distribution and clustering of CAF subtypes in pancreas tumor tissue

**Fig. 2.**
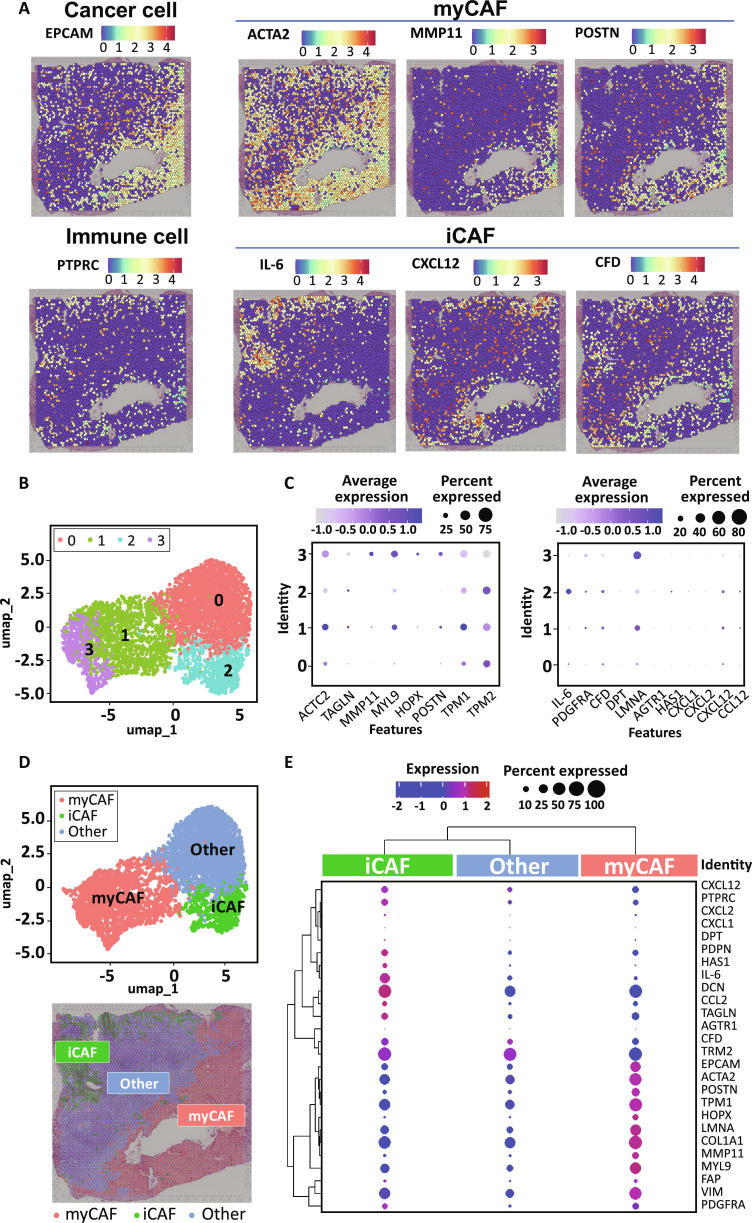
Identification of the heterogeneity of myCAF and iCAF in PDAC through spatial transcriptomics on human PDAC tissue. (A) Distribution of cancer cells (EpCAM^+^), immune cells (CD45^+^), myCAF (ACTA2^+^, MMP11^+^, POSTN^+^), and iCAF (IL6^+^, CXCL12^+^, CFD^+^) based on cell subtype-specific gene expression. (B) Unsupervised clustering of spatial transcriptomics data. (C) Comparative expression analysis of representative myCAF- and iCAF-associated genes across clusters. (D) Clustering of myCAF, iCAF, and other cell populations, along with their spatial localization within the tumor tissue. (E) Comparison of the expression levels of myCAF- and iCAF-specific genes among myCAF, iCAF, and other groups.

To investigate the various fibroblast subtypes present in the pancreatic TME and their spatial distribution, we performed transcriptomics using tumor tissues from patients with pancreatic cancer. First, we analyzed the distribution of tumor (EpCAM^+^) and immune (PTPRC/CD45^+^) cells within the tumor tissue, confirming that these 2 cell populations were largely nonoverlapping (Fig. 2A). We subsequently examined the spatial distribution of CAF subtypes by utilizing representative markers: myCAF (ACTA2, MMP11, POSTN) and iCAF (IL6, CXCL12, CFD) (Fig. 2A). Our analysis revealed that myCAFs were primarily localized near the tumor cells, whereas iCAFs exhibited a distribution pattern similar to that of immune cells. To further delineate CAF subtypes within the tumor tissue, we performed unsupervised clustering, categorizing all spatial transcriptomic spots into 4 distinct clusters (Fig. 2B). DEG analysis across these clusters revealed that clusters 1 and 3 were enriched for myCAF-associated genes (ACTA2, MYL9, POSTN, TPM1), while cluster 2 exhibited high expression of iCAF-associated genes (IL6, CFD, CXCL12, CCL2) (Fig. 2C). Thus, we classified clusters 1 and 3 as myCAF, cluster 2 as iCAF, and cluster 0 as “other”, and visualized these classifications using UMAP and spatial mapping within the tumor tissue (Fig. 2D). Furthermore, comparative gene expression analysis between the CAF subtype-specific clusters demonstrated that iCAF and myCAF were well-separated based on distinct gene signatures. Our findings highlight that the 2 major CAF subtypes, myCAF and iCAF, are spatially distinct within TME, particularly regarding tumor and immune cell localization. Finally, by identifying a set of subtype-specific gene signatures, we established a robust classification of myCAF, iCAF, and “other” fibroblast populations, laying the groundwork for further in-depth molecular characterization of these CAF subtypes.

### Transcriptomic and functional differentiation of iCAF and myCAF in PDAC

**Fig. 3.**
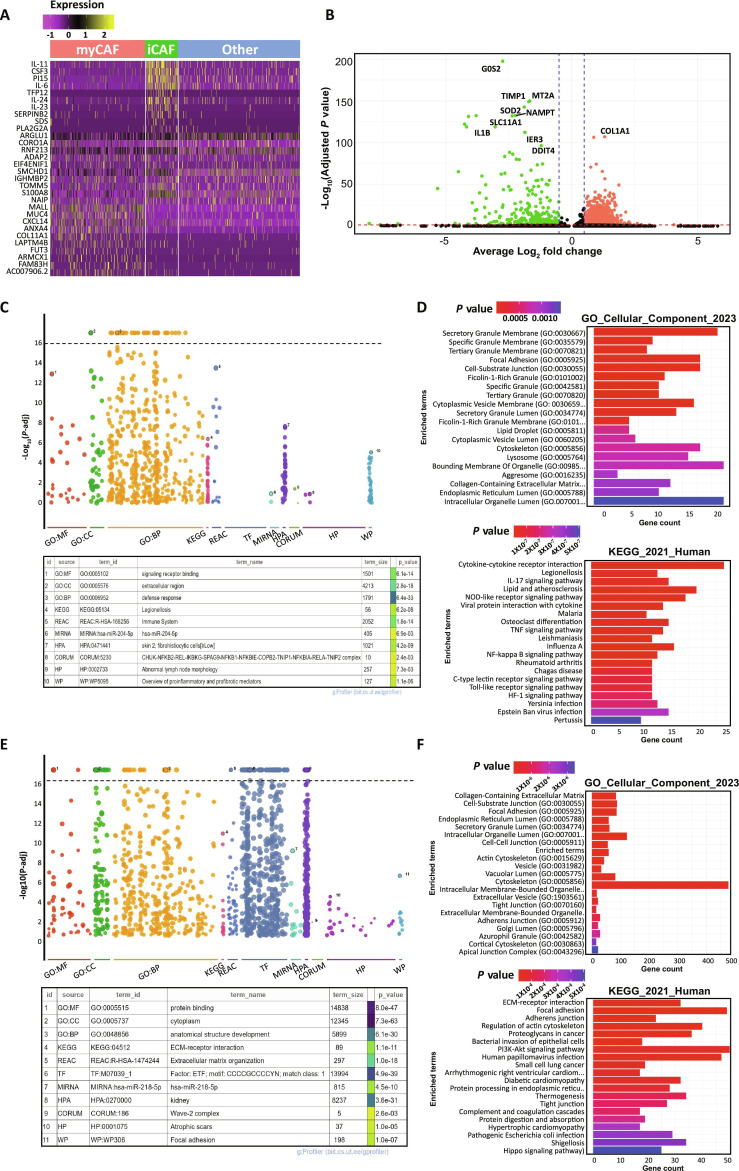
RNA expression level and the number of gene counts of CAFs for the extraction of molecular and functional characteristics using RNA sequencing results. (A) Heatmap of RNA expression level of iCAF, myCAF, and other groups in tumor tissues from pancreatic cancer patients. (B) Volcano plot with log_2_ fold for iCAF and myCAF showing high expressed gene in each group (C and E) shows the expression of cell markers for iCAF and myCAF, respectively, through dot plot. (D and F) Functional analysis of iCAF and myCAF through gene ontology. *P* value ranges indicated within the graph.

We used heatmaps and volcano plots to visualize gene expression levels in iCAF and myCAF and other groups using transcriptomic sequencing results. In Fig. 3A, yellow-colored portions are highly expressed RNA, whereas dark purple portions are relatively low expressed RNA. In the case of myCAF, high-expression RNA is mainly MALL, MUC4, CXCL14, and ANXA4, and whereas iCAF shows highly expressed genes such as IL11, CSF3, PI15, and IL6 as inflammatory relative markers. For the result of volcano plots with RNA-seq, we identified significant genes based on CAF subtypes, with a notable increase in gene expression particularly in iCAFs. Among these, the expression levels of G0S2, MTA2A, NAMPT, SOD2, SLC11A, TIMP1, and IL1B were significantly up-regulated (Fig. 3B). G0S2 is a regulator of lipid metabolism [19], while MTA2A has been reported to induce the expression and secretion of insulin-like growth factor binding protein 2 (IGFBP2) [20]. NAMPT plays a crucial role in maintaining zinc and copper homeostasis for DNA replication [21]. On the other hand, in myCAF, relatively up-regulated gene is collagen type 1α1 (COL1A1), which plays an essential role in inducing the differentiation and metastasis of cancer cells [22] and promotes tumor infiltration [23]. Collagen gene associated with ECM is prominently up-regulated in myCAF, and inflammatory interleukin genes such as IL1B are expressed in accordance with the characteristics of iCAF. Thus, the subtype of CAF at the gene expression level shows significant differences as their roles in TME. Figure 3C and E shows the dot plot and are the results of the gene ontology to find out the gene function. For gene ontology, GO, KEGG, REAC, TF, MIRNA, HPA, CORUM, HP, and WP were used for bioinformatic resources. In iCAFs, functional analysis of individual libraries revealed strong associations with signaling receptor binding, extracellular region, defense response, and immune system processes. Further analysis of cellular components identified significant correlations with various granule and lipid-related components, including secretory granule membrane, cytoplasmic vesicle membrane, and lipid droplets. Additionally, pathway and disease enrichment analysis predicted associations with cytokine–cytokine receptor interactions, IL-17 signaling pathway, and lipid metabolism/atherosclerosis (Fig. 3C and D).

In contrast, myCAFs exhibited strong associations with protein binding, cytoplasm, ECM–receptor interactions, and ECM organization. Further cellular component analysis highlighted significant correlations with collagen-containing ECM and focal adhesion, while pathway and disease enrichment analysis indicated links to ECM–receptor interactions, focal adhesion, and regulation of the actin cytoskeleton (Fig. 3E and F). In summary, iCAFs are characterized by functions related to vesicle- and lipid-associated components and pathways, whereas myCAFs are primarily involved in collagen-rich ECM interactions and adhesion-related processes. These distinct functional characteristics of CAF subtypes contribute to the complexity of the TME, potentially influencing immune evasion and therapeutic resistance.

### Raman spectra acquisition of HPaSC-induced iCAFs and myCAFs for molecular profiling

**Fig. 4.**
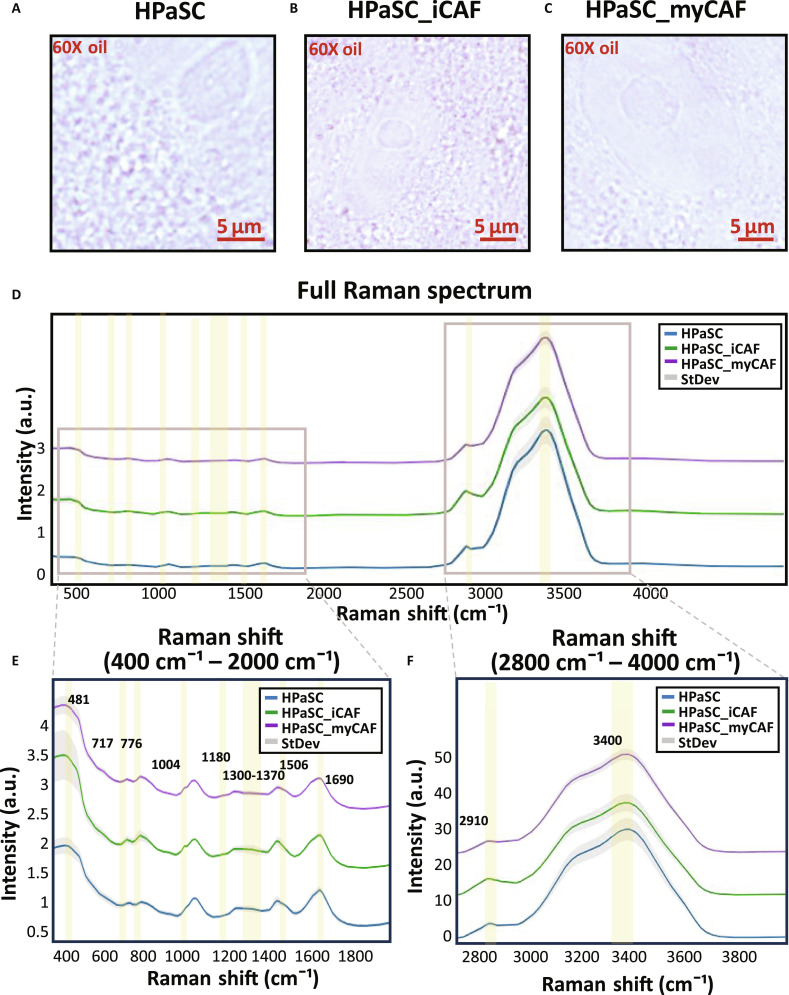
Raman spectral comparison for molecular profiling HPaSC-derived CAF subtypes. Bright-field images of (A) HPaSC (*n* = 8), (B) HPaSC_iCAF (*n* = 10), and (C) HPaSC_myCAF (*n* = 12) are captured by 60× oil immersion objective lens (scale bar, 5 μm) along with Raman spectrum acquirement. (D) Full Raman spectrum. (E) Mean Raman spectrum of 3 groups (HPaSC: blue, iCAF: green, myCAF: purple) as solid line with standard deviation. Shaded line is visualized between Raman shift ranging from 400 to 2,000 cm^−1^ and (F) Raman shift ranging from 2,800 to 4,000 cm^−1^ showing variations. Raman spectrum was normalized at 1,040 cm^−1^, which is the PFA signal.

We acquired Raman spectra of iCAFs, myCAFs that are induced from HPaSC and HPaSC as control, after 4% PFA fixation. The acquisitions of Raman spectrum from each cell subtype are shown in Fig. 4A to C as bright-field images. Through simple morphological feature comparison, iCAFs and myCAFs showed clear nuclear boundaries compared with HPaSC, while the size of nucleus is reduced. This suggests that phenotypic transitions may occur upon differentiation into each subtype. For detailed molecular fingerprints, we normalized Raman spectrum at 1,040 cm^−1^. which is the PFA signal. We obtained full spectrum ranging from 300 to 5,000 cm^−1^ shown in Fig. 4D. We focused on the Raman shifts ranging from 400 to 2,000 cm^−1^ in Fig. 4E. This range is the fingerprint region enriched with nucleic acids, proteins, amino acids, and lipids with various bending, stitching, and ring breath vibration detection [24]. We also focused on the region above 2,800 cm^−1^ as shown in Fig. 4F for lipid cell membrane with symmetric/antisymmetric stretching of CH. However, the range from 1,800 to 2,800 cm−¹ is considered a Raman-silent region, as it contains no strong Raman signals from biological molecules [25]. Raman spectrum ranging from 400 to 2,000 cm^−1^ shows high variation in Raman shift intensity for iCAFs compared with HPaSC and myCAFs. Notably, the Raman peaks between 700 and 800 cm^−1^ and 1,200–1,400 cm^−1^, as shown in Fig. 4E, showed larger standard deviations in iCAFs. These ranges contain DNA ring breathing mode, amide III band, and lipid-related peaks. The considerable diversity in Raman intensity for iCAFs suggests biochemical heterogeneity, reflecting diverse metabolic state or functional variability such as inflammatory activation. All featured Raman spectrum assignment is listed in Table 1.

**Table 1.** Assignment of Raman shift

Raman shift (cm^−1^)	Assignment	Ref.
481	DNA	[34]
680	Guanine	[35]
717	Choline group, phospholipids head	[36]
776	Phosphatidylinositol	[36]
830	Stretch of nucleic acid	[37]
992	Carbone ring, benzene	[38]
1,004	Phenylalanine	[39]
1,040	Paraformaldehyde	[16]
1,175	Cytosine, guanine	[40]
1,180	CH_3_/CH_2_ twisting or bending mode of lipid/collagen	[41]
1,300	Fatty acid, CH deformation	[42]
1,370	dATP	[35]
1,423	NH in plane deformation	[43]
1,506	Cytosine	[44]
1,638	Amide I	[45]
1,690	C=O stretching, amide I	[46]
1,700	Amide I	[47]
2,811	CH stretching vibration	[48]
2,896	CH_2_ symmetric vibrations, lipid	[49]
2,910	CH_3_ stretching vibrations	[49]
2,950	CH vibration	[49]
3,400	OH stretch	[50]

### Application of machine learning algorithm on Raman spectra for discriminating iCAFs and myCAFs

**Fig. 5.**
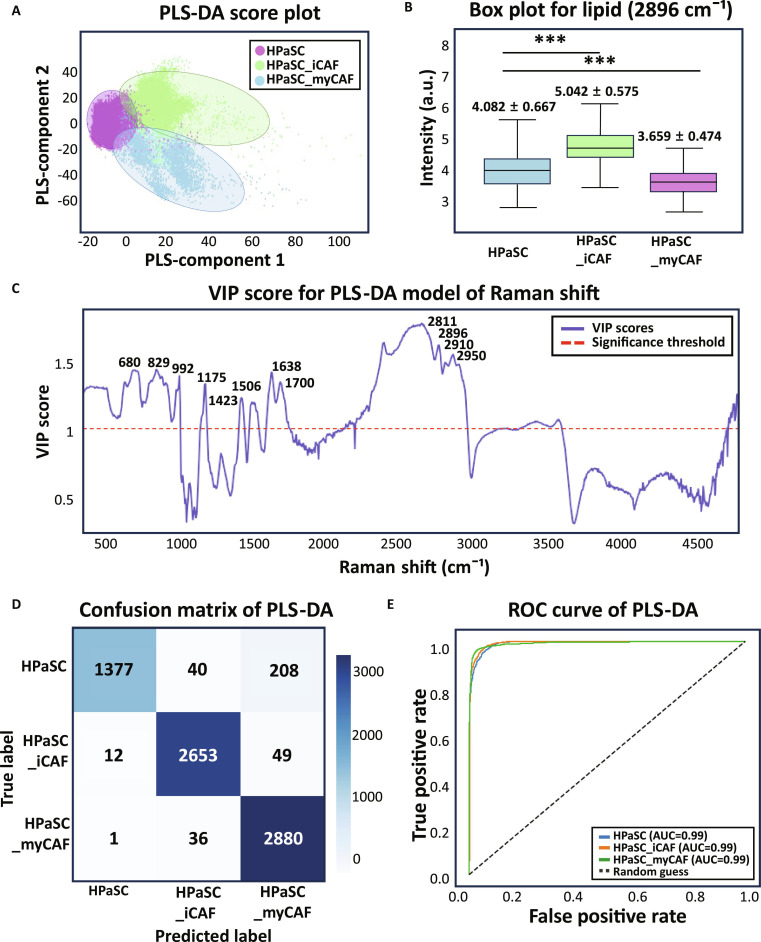
Raman spectral analysis for classification of CAF subtypes derived from HPaSC using PLS-DA. (A) Raman spectrum distribution as the result of PLS-DA shows clear separation between HPaSC (purple), iCAF (green), and myCAF (blue) with the indication of dashed oval. (B) Box plots for specific Raman shifts for lipid signal (2,896 cm^−1^) showed strong intensity in iCAF among the group with the indication of statistically significant differences (***P < 0.05). (C) VIP score, with dashed line indicating significance threshold (VIP score = 1.0), highlights key Raman peaks contributing to class separation (D). Confusion matrix on the test set (0.3) shows high accuracy across all classes. (E) ROC curves with area under the curve (AUC) values of 0.99 show excellent diagnostic performance.

The normalized Raman spectrum at PFA peak is then separated under the application of PLS-DA. PLS-DA is supervised classification algorithm commonly employed in Raman spectrum classification with the partial least square regression. Through this process, VIP score can be calculated to identify the most contributing Raman shifts for the class separation with the significant threshold value of 1.0. The significant features were compared between HPaSC, HPaSC_iCAFs, and HPaSC_myCAFs, highlighting molecular differences in various biochemical changes. In Fig. 5A, the data distribution is shown after PLS-DA with application of PLS regression component = 2 on the entire Raman spectrum for each group. Clear data separation is marked with dashed oval on HPaSC (purple), HPaSC_iCAF (green), and HPaSC_myCAF (blue) data in the scatterplot graph. This reflects that Raman signals can be clearly separated. To find out which Raman shift contributes to the class separation, we extract VIP scores from PLS-DA in Fig. 5C. We focused on high VIP score above the threshold of 1.0, and the main peaks were 481 (DNA), 680 (guanine), 830 (stretch of nucleic acid), 992 (carbon ring, benzene), 1,175 (cytosine, guanine), 1,423 (NH in plane deformation), 1,506 (cytosine), 1,638 (amide I), 1,700 (amide I), 2,811 (CH stretching vibration), 2,896 (CH_2_ symmetric vibrations, lipid), 2,910 (CH_3_ stretching vibrations), and 2,950 cm^−1^ (CH vibration). For the comparison of the intensity of Raman peak extracted from VIP score, we performed box plot analysis of Raman peak intensity. Notably, 2,896 cm^−1^ associated with lipid signal showed increase in HPaSC_iCAFs shown in Fig. 5B as box plot. For each group, the mean intensity value and standard deviation were 4.082 ± 0.667 for HPaSC, 5.042 ± 0.575 for HPaSC_iCAFs, which was the highest, and 3.659 ± 0.474 for HPaSC_myCAFs, which was the lowest. The corresponding percentages of standard deviation were 16.35%, 11.41%, and 12.96%, respectively. Among Raman shifts with VIP score over 1.0, iCAF also showed strong intensity in DNA-related peak at 481 cm^−1^ and lipid-related signal at 2,811 cm^−1^ as shown in Fig. S1. However, high Raman shift intensity of myCAFs appeared in 680 cm^−1^, which is the guanine-related signal shown in Fig. S2. Raman peaks at 2,910 and 2,950 cm^−1^, which are lipid-related signals in Fig. S3, show stronger intensity for iCAFs. Although the Raman intensity in myCAFs was generally lower than in iCAFs, myCAFs have elevated intensity compared with HPaSC at 481, 830, 922, and 1,175 cm^−1^ shown in Figs. S2 and S3. These Raman signals are related nucleotide signal that can assume the DNA or RNA expression increase in myCAFs. On the other hand, HPaSC showed high intensity at 1,638 and 1,700 cm^−1^, which are both amide I-related signals. Since both iCAFs and myCAFs had lowered intensity at these 2 Raman shifts, protein structural formation may have changed from HPaSC.

To test classification performance, we conducted confusion matrix and ROC curve analysis. PLS-DA classification model on Raman spectrum can accurately distinguish among HPaSC, iCAF, and myCAF subtypes. We split the data into training set:test set = 7:3 ratio. As a result, iCAFs and myCAFs were correctly classified in 2,653 and 2,880 cases, respectively. HPaSC showed 1,377 correct predictions. There are some misclassifications of myCAFs, indicating partial spectral similarity between HPaSC and myCAFs. The ROC curve analysis yielded an AUC of 0.99 for all classes, demonstrating high sensitivity and specificity of the model. These results highlight the capability of PLS-DA to precisely discriminate CAF subtypes based on their molecular features, and support the potential of label-free Raman spectrum classification platforms on CAFs. All calculation results derived from confusion matrix is summarized in Table 2. To qualify the robustness of the PLS-DA model, 5-fold cross-validation (CV) with 70% training set and 30% test set split of the entire dataset was performed, where each fold is used once as an independent test set. The model achieved a mean accuracy across folds of 0.9368, and the detail of classification performance (mean ± SD for 5 folds) is summarized in Table S1.

**Table 2.** Classification accuracy table

Class	Precision	Recall	F1-score	Accuracy
HPaSC	0.9906	0.8474	0.9134	
HPaSC_iCAF	0.9722	0.9775	0.9748	
HPaSC_myCAF	0.9181	0.9873	0.9514	
Overall				0.9524

### Lipid metabolomics for validating Raman spectrum of CAFs

**Fig. 6.**
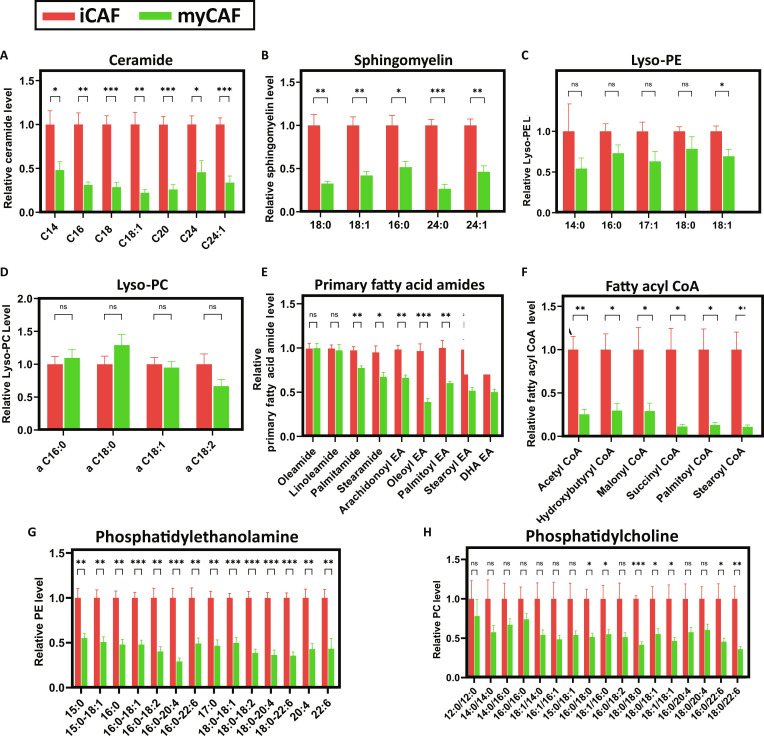
Comprehensive lipidomic profiling and lipid alteration for iCAF and myCAF. HPaSCs as control group were treated by IL-1α and TGF-β1, which induce the phenotype of iCAF (*n* = 3) and myCAF (*n* = 3), respectively. Each graph shows the relative alteration of (A) ceramide, (B) sphingomyelin, (C) Lyso-PE, (D) Lyso-PC, (E) primary fatty acid amides, (F) fatty acyl CoA, (G) PE, and (H) PC (*P < 0.05, **P < 0.01, ***P < 0.001).

As a result of molecular profiling from the Raman spectrum, the main peak contributing to the subtype separation is a lipid-related signal (Fig. 5B). In the colorectal cancer, CAFs undergo lipid reprogramming to control the progression of colon cancer [26]. In PDAC, characterized by desmoplasia and nutrient and oxygen scarcity, CAFs transfer nutrients to the cancer cells through lipid metabolism [27]. Lipid is a primary fuel source and signaling molecule for cancer-related growth. Therefore, lipidomic analysis was performed, and as in previous study, CAF subtypes (iCAFs and myCAFs) were induced from HPaSC by IL-1α and TGF-β1, respectively. Using CAF subtype-specific cell lines, we conducted a quantitative comparative analysis of lipid metabolites using cell lysates (Fig. 6 and Fig. S4). First, in the ceramide family (C14, C16, C18, and others) and the sphingomyelin family (18:0, 18:1, 16:0, and others), quantitative analysis revealed a significant increase in nearly all metabolites in the iCAF phenotype (Fig. 6A and B). Similarly, PE and Lyso-PE metabolites, including 15:0, 16:0, 17:0, and 20:4, exhibited higher levels in iCAFs (Fig. 6C and G). This up-regulation pattern was also observed in PC, Lyso-PC, and primary fatty acid amides. Notably, fatty acyl-CoA levels were elevated 3- to 4-fold in iCAFs (Fig. 6E and H). Changes in lipid metabolism can affect cell growth, proliferation, mobility, autophagy, and apoptosis in many cancers [28,29]. Pancreatic stellate cell (PSC)-derived CAFs secrete several metabolites, including PE, Lyso-PE, PC, and Lyso-PCs. Among them, Lyso-PC induces cell proliferation or migration of PDAC, leading to the aggressive cancer characteristics of PDAC [30]. CAFs abundantly secrete Lyso-PC, which PDAC cells uptake and convert into PC to maintain membrane integrity [27]. Additionally, in vivo experiments suggest that CAFs experience less hypoxia than that of malignant tumor cells and may serve as a potential lipid source for PDAC cells [27]. Since lipid-related signals exhibited significant differences in Raman spectra of CAFs, lipid analysis was conducted to identify the types of lipids or fatty acid amides predominantly metabolized in each subtype. Overall, iCAF-phenotype cells exhibited increased levels of lipid metabolites across most categories, demonstrating a significant metabolic distinction from myCAFs. These metabolic differences suggest that the 2 CAF subtypes may play distinct roles within the TME, potentially influencing tumor cell growth and migration, immune cell infiltration and activation, ECM, vascular formation, as well as drug responsiveness and resistance [31].

## Discussion

Our study demonstrates a label-free Raman spectroscopic approach to distinguish CAF subtypes based on their chemical composition. Combining Raman spectrum of cells with machine learning algorithm, CAF subtype derived from HPaSC can be distinguished with 95% accuracy. Raman spectroscopy already shows a significant potential in cancerous and noncancerous cells in previous studies, revealing chemical composition and characteristics of breast cancer cells compared to noncancerous cells [32]. Since it is challenging to approach proper treatment in PDAC with various subtypes of CAFs within TME showing different response to therapeutic agents, determination of CAF subtypes is crucial for optimizing treatment strategies. We acquired Raman spectrum from cultured iCAFs and myCAFs derived from HPaSC to reveal their intrinsic chemical composition of each subtype without any biomarkers or fluorescence marker.

For the validation of Raman spectrum fingerprint, we compared the CAFs in DNA/RNA levels to identify which genes were predominant in human PDAC tissue. As a result, iCAFs show high expression of lipid metabolism-related gene, while myCAFs exhibit up-regulation of collagen-related genes associated with the ECM. Since the CAFs shows clear differences in RNA gene expression level, iCAFs and myCAFs each have their distinct roles within the TME. Furthermore, spatial transcriptomics on human PDAC tissue revealed that iCAFs using gene markers IL6 and CXCL12 and myCAFs using gene markers ACTA2 and MMP11 were spatially distributed and correlate with tumor and immune cell localizations. iCAFs and myCAFs exist in spatially different area within the PDAC tissues and express different types of genes reflecting their functional roles.

Thus, using Raman spectra analysis to examine the chemical composition different at the molecular level can classify iCAFs and myCAFs derived from HPaSC since they both show significant differences in gene expression level and spatially different distribution within PDAC. In particular, Raman spectrum of iCAFs exhibited Raman intensity variation at several Raman shift regions roughly at 700 to 800 cm^−1^ and 1,200 to 1,400 cm^−1^, while Raman shifts for HPaSC and myCAFs are stable with less standard variation shown in Fig. 4E. Since metabolic changes in cells and subtypes indicate various Raman spectrum intensity, iCAFs and myCAFs from HPaSC show significantly different Raman peaks and this can be classified using PLS-DA (PLS regression component = 2). The results of PLS-DA were visualized as scatterplot, and these 3 groups of HPaSC, iCAFs, and myCAFs were clearly separated with their spectra features. Notably, peaks associated with lipid-related variations including the bands near 2,896, 2,910, and 2,950 cm^−1^ were the influential spectra according to the VIP score. These results matched with the transcriptomics analysis that iCAFs have high expression in lipid metabolic genes. To evaluate the classification performance, we constructed a confusion matrix with 70% training set and 30% test set and calculated ROC curve based on test data. As a result, we reached 95% accuracy in confusion matrix and 0.99 for AUC from ROC curve.

We sequentially conducted quantitative lipidomic analysis to support Raman spectral observation and further validate the distinct metabolic characteristics of CAF subtypes. Thie results demonstrated a significant increase in various lipids in iCAFs compared with including ceramide family (C14 to C18), sphingomyelin family (18:0, 18:1, 16:0), PE, and Lyso-PE. These findings confirm that iCAFs undergo lipid-enriched metabolic reprogramming, reinforcing the Raman-based evidence that iCAFs and myCAFs are metabolically distinct. The results of lipidomic analysis that correlated to the results of Raman peak comparison are the evidence of the previous findings since lipid metabolism alterations are related to cancer growth, motility, and cell apoptosis [33]. However, we used cultured iCAFs and myCAFs induced from HPaSC for Raman analysis and lipidomic analysis, while spatial transcriptomics analysis was conducted using human PDAC tissue, which provides deeper insights into gene expression and functional diversity within the complex and highly structured TME. The dynamics and interactive components between CAFs and TME may not fully represent HPaSC-derived iCAFs and myCAFs.

For example, collagen-related peaks that are typically associated with myCAFs were not clearly observed in our dataset compared with the transcriptomic data. Instead, Raman peak at 680 cm^−1^ associated with guanine is slightly higher in myCAFs compared with HPaSC and iCAFs. The absence of pronounced collagen-associated Raman peaks in induced myCAF cells may reflect a limitation of cultured cell, since our transcriptomic data were obtained from human PDAC tissue. Nevertheless, we were able to correlate the important spectra features of iCAFs corresponding with gene expression pattern identified through transcriptomics with lipid-related signals. Combining these results with machine learning algorithm such as PLS-DA, we could reach high classification accuracy for iCAFs and myCAFs as well as the Raman spectra differences through VIP score, demonstrating the coherent alignment between molecular signals to the gene characteristics of iCAFs. Based on this work frame, it is adaptable and can be scaled to account for broader CAF diversity, including intermediate and mixed phenotypes that coexist in the TME.

Lastly, this study is limited by the use of a single commercially available HPaSC line, which may restrict the generalizability of our findings. Nevertheless, this uniform origin enabled a clearer comparison between iCAFs and myCAFs. Future studies using CAFs from diverse donors and clinical samples will be required to validate and extend these observations. Importantly, this approach may also provide a foundation for developing label-free diagnostic platforms or cell-profiling technologies to support personalized therapeutic strategies in pancreatic cancer. Moreover, our framework is adaptable and can be scaled to account for broader CAF diversity, including intermediate and mixed phenotypes that coexist in the TME.

This study aimed to characterize the biological and chemical differences between myCAFs and iCAFs. Using label-free Raman spectroscopy integrated with machine learning algorithm, we identified distinct Raman peaks for CAF subtype, iCAFs, and myCAFs derived from HPaSC. Multivariate analysis with PLS-DA revealed significant spectral distinctions existing in each CAF subtype. Particularly, elevated lipid-associated Raman shift intensity appeared in iCAFs. In the meantime, myCAFs showed strong intensity at guanine signal among the 3 groups. myCAFs also showed higher intensity for DNA-related peak compared to HPaSC, which can be a transcriptional change between HPaSC and myCAFs. From this Raman intensity difference, the CAFs were classified with 95% accuracy. Lipid-associated Raman features for iCAFs were strongly validated through quantitative lipidomic profiling including ceramide, sphingomyelins, and phosphatidyl-ethanolamine. This result supports the spatial transcriptomics analysis of human PDAC tissue, confirming the distinct molecular identities of each CAF subtype. Multiple spectra were acquired per cell, and the spectrum level spilt may include some spectra that appeared in both the training and test folds. This procedure may yield an upper bound estimate. However, Raman spectra at the subcellular level within heterogeneous intracellular space of a cell are not identical, and each spectrum from different CAFs still remains for informative feature discovery to support CAF-subtype discrimination. This approach using label-free Raman spectroscopy integrated with machine learning establishes the foundation for future noninvasive classification platforms for therapeutic development targeting specific CAF subtypes in pancreatic cancer.

## Data Availability

Data are available from the corresponding author upon request.
