# Supplementary material for: Molecular Profiling of Inflammatory and Myofibroblast Cancer-Associated Fibroblast Subtypes Derived from Human Pancreatic Stellate Cells Using Machine Learning-Based Label-Free Raman Spectroscopy
Source: Biomater Res. 2025 Dec 9;29:0292. doi: 10.34133/bmr.0292 (PMC12686345; doi:10.34133/bmr.0292)
Supplement: Supplementary 1 — Figs. S1 to S4 Table S1 [file bmr.0292.f1.zip › CAF_Raman_Supplement_Final_Second_revision.docx]

**Molecular Profiling of Inflammatory and Myofibroblast Cancer-Associated Fibroblast (CAF) Subtypes Derived from Human Pancreatic Stellate Cells Using Machine Learning-Based Label-Free Raman Spectroscopy**

Minju Cho^1, †^, Eun-Young Koh^1, †^, Yeounhee Kim^1^, Seong-Jin Kim^1^, Chan-Gi Pack^2^,

Eunsung Jun^1,4, *^, Jun Ki Kim^1,3, *^

^1^ Department of Convergence Medicine, Brain Korea 21 Project, University of Ulsan, College of Medicine, Asan Medical Center, Seoul, Republic of Korea

^2^ Department of Biomedical Engineering, University of Ulsan College of Medicine, Asan Medical Center, Seoul, Republic of Korea

^3^ Biomedical Engineering Research Center, Asan Medical Center, Seoul, Republic of Korea

^4^ Division of Hepato-Biliary and Pancreatic Surgery, Department of Surgery, University of Ulsan College of Medicine, Asan Medical Center, Seoul, Republic of Korea

† These authors contributed equally to this work.

***Correspondence**

Eunsung Jun, MD. PhD

Email: [eunsungjun@amc.seoul.kr](mailto:eunsungjun@amc.seoul.kr)

Jun Ki Kim, PhD

Email: [kim@amc.seoul.kr](mailto:kim@amc.seoul.kr)

Number of pages: 6

Number of figures: 4

Number of schemes: 0

Number of tables: 1

| **Figure S1** | Summarized boxplot of Raman intensity comparison at 1423,1506,1683,1700 and 2811 cm⁻¹ |
| --- | --- |
| **Figure S2** | Summarized boxplot of Raman intensity comparison at 680, 829, 992, and 1175 cm⁻¹ |
| **Figure S3** | Summarized boxplot of Raman intensity comparison at 481, 2910, and 2950 cm⁻¹ |
| **Figure S4** | The raw data of lipidomic profiling and lipid alternation for iCAF and myCAF |
| **Table S1** | Summary of 5-fold cross validation (Mean ± SD |


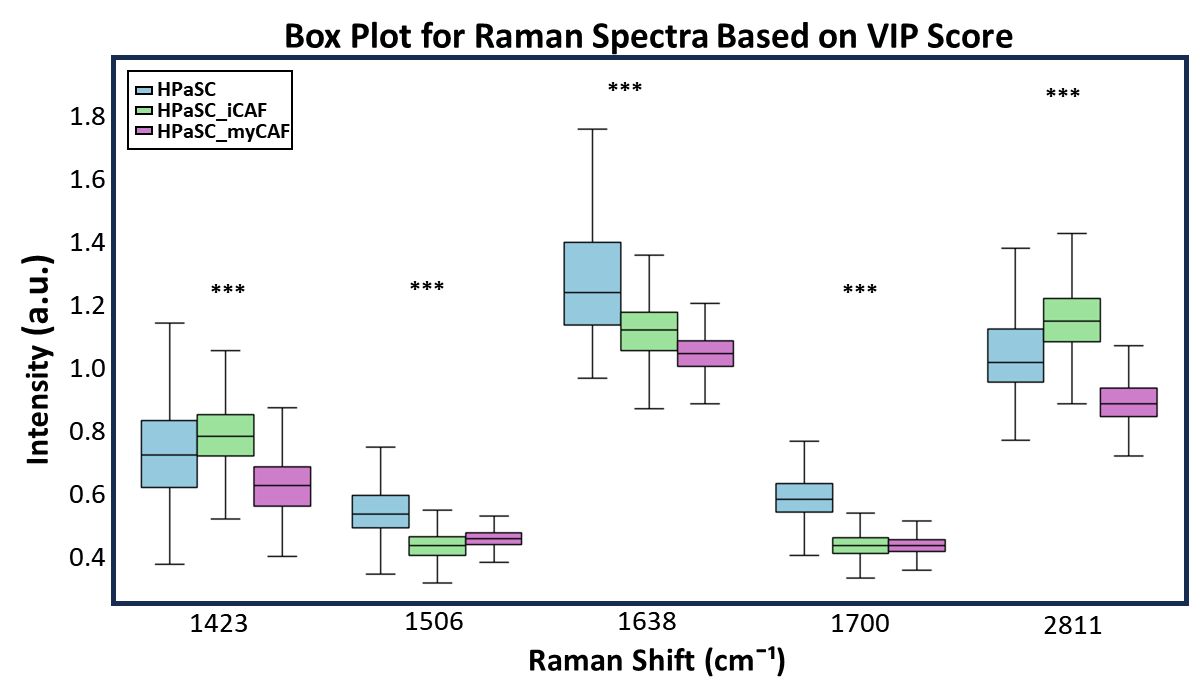


**Figure S1.** Summarized boxplot of Raman intensity comparison for HPasC (sky blue) , iCAF (green) and myCAF (myCAF) at 1423 (NH in plane deformation), 1506 (cytosine), 1638 (Amide I), 1700 (Amide I), 2811 cm⁻¹ (CH stretching vibration). Statistical significance is indicated above the box plot (*** P<0.001).


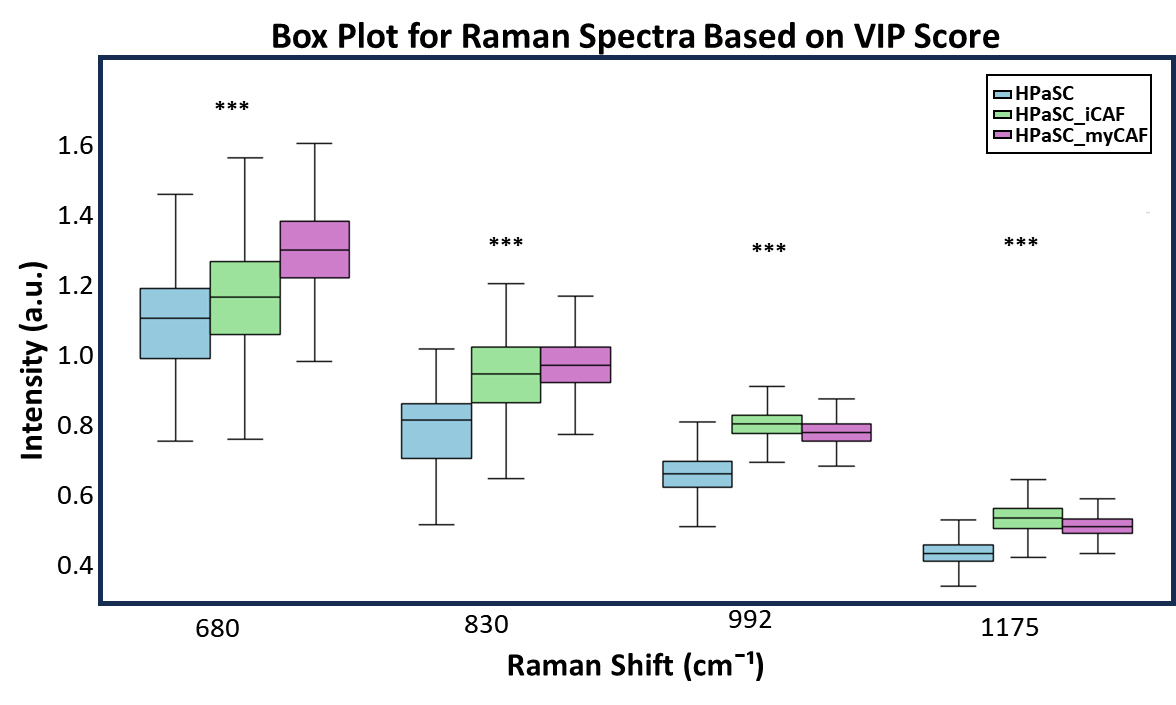


**Figure S2.** Summarized boxplot of Raman intensity comparison for HPasC (sky blue), iCAF (green) and myCAF (myCAF) at 680 (Guanine), 830 (nucleic acid), 992 (Carbone ring, Benzene), 1175 (Cytosine, Guanine). Statistical significance is indicated above the box plot (*** P<0.001).


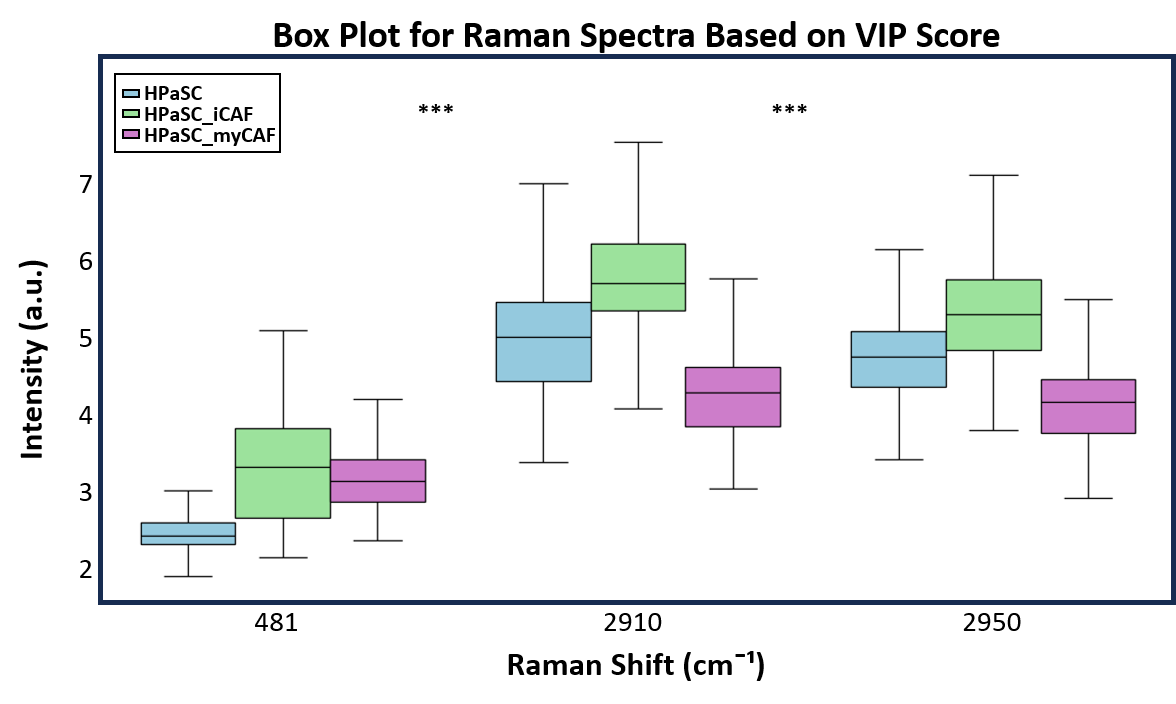


**Figure S3.** Summarized boxplot of Raman intensity comparison for HPasC (sky blue), iCAF (green) and myCAF (myCAF) at 481 (DNA), 2910 (CH_3_ stretching vibration), and 2950 cm⁻¹ (CH vibration). Statistical significance is indicated above the box plot (*** P<0.001).


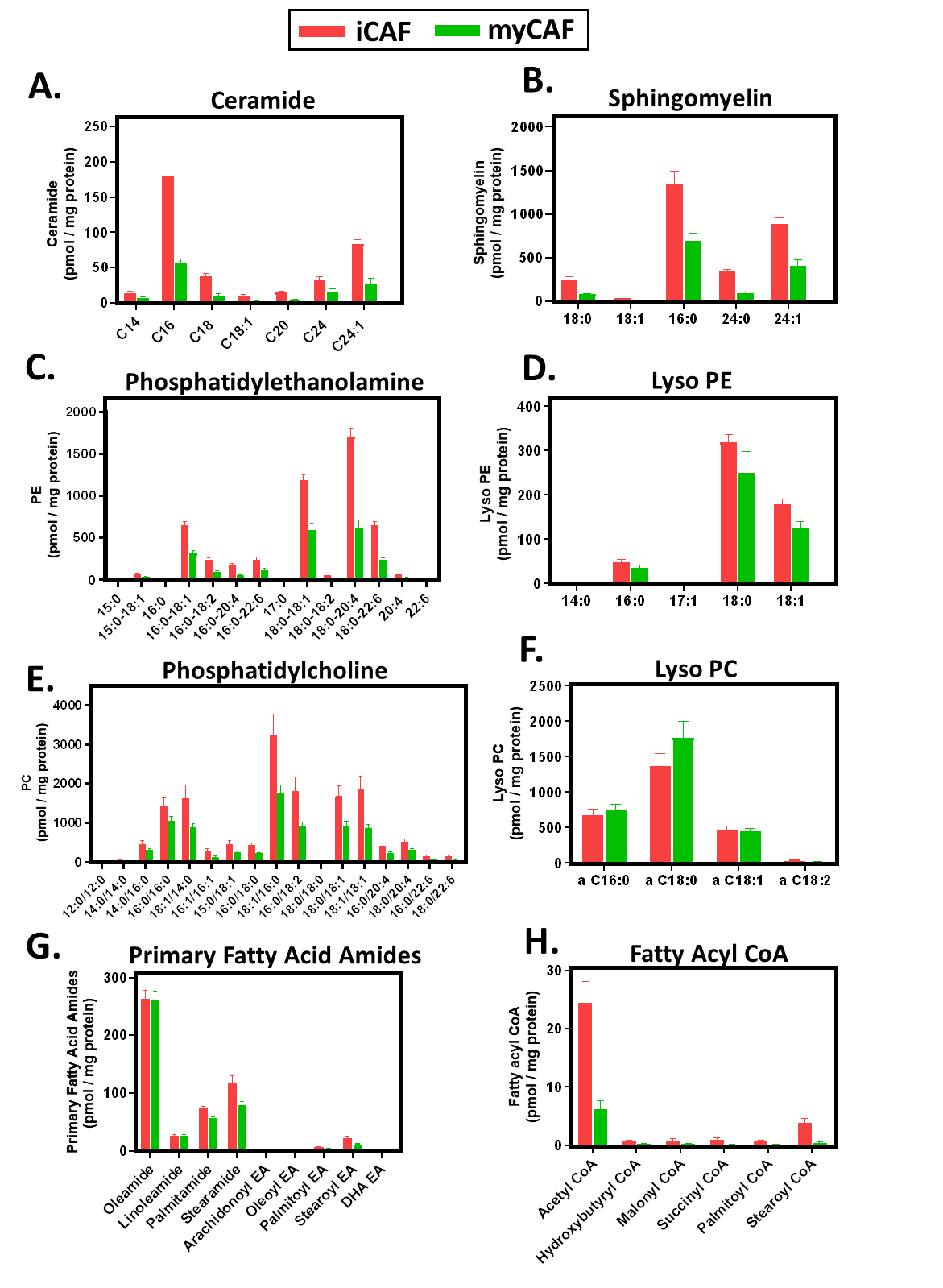


**Figure S4.** The raw data of lipidomic profiling and lipid alternation for iCAF and myCAF. HPaSC as control group were treated by IL-1a and TGF-b1 which induce the phenotype of iCAF (n=3) and myCAF (n=3) respectively. Each graph shows the alternation of (A) ceramide, (B) sphingomyelin, (C) phosphatidylethanolamine (PE), (D) Lyso PE, (E) phosphatidylcholine (PC) , (F) Lyso PC, (G) primary fatty acid amides, and (H) Fatty acyl CoA. (* p<0.05, ** p<0.01, *** p<0.001**)**

**Table S1.** 5-fold classification report (Mean ± SD)

| **Class** | **Precision** | **Recall** | | **F1** | | **Accuracy*** |
| --- | --- | --- | --- | --- | --- | --- |
| **CAF** | 0.9815 ± 0.0030 | 0.7170 ± 0.0145 | | 0.8286 ± 0.0105 | |  |
| **iCAF** | 0.9451 ± 0.0025 | 0.9814 ± 0.0017 | | 0.9629 ± 0.0020 | |  |
| **myCAF** | 0.9181 ± 0.0032 | 0.9857 ± 0.0023 | | 0.9507 ± 0.0017 | |  |
|  |  | |  | |  | **0.9368 ± 0.0028** |
