# Supplementary material for: Molecular Profiling of Inflammatory and Myofibroblast Cancer-Associated Fibroblast Subtypes Derived from Human Pancreatic Stellate Cells Using Machine Learning-Based Label-Free Raman Spectroscopy
Source: Biomater Res. 2025 Dec 9;29:0292. doi: 10.34133/bmr.0292 (PMC12686345; doi:10.34133/bmr.0292)
Supplement: Supplementary 1 — Figs. S1 to S4 Table S1 [file bmr.0292.f1.zip › Figure_S4_PDF.pdf]

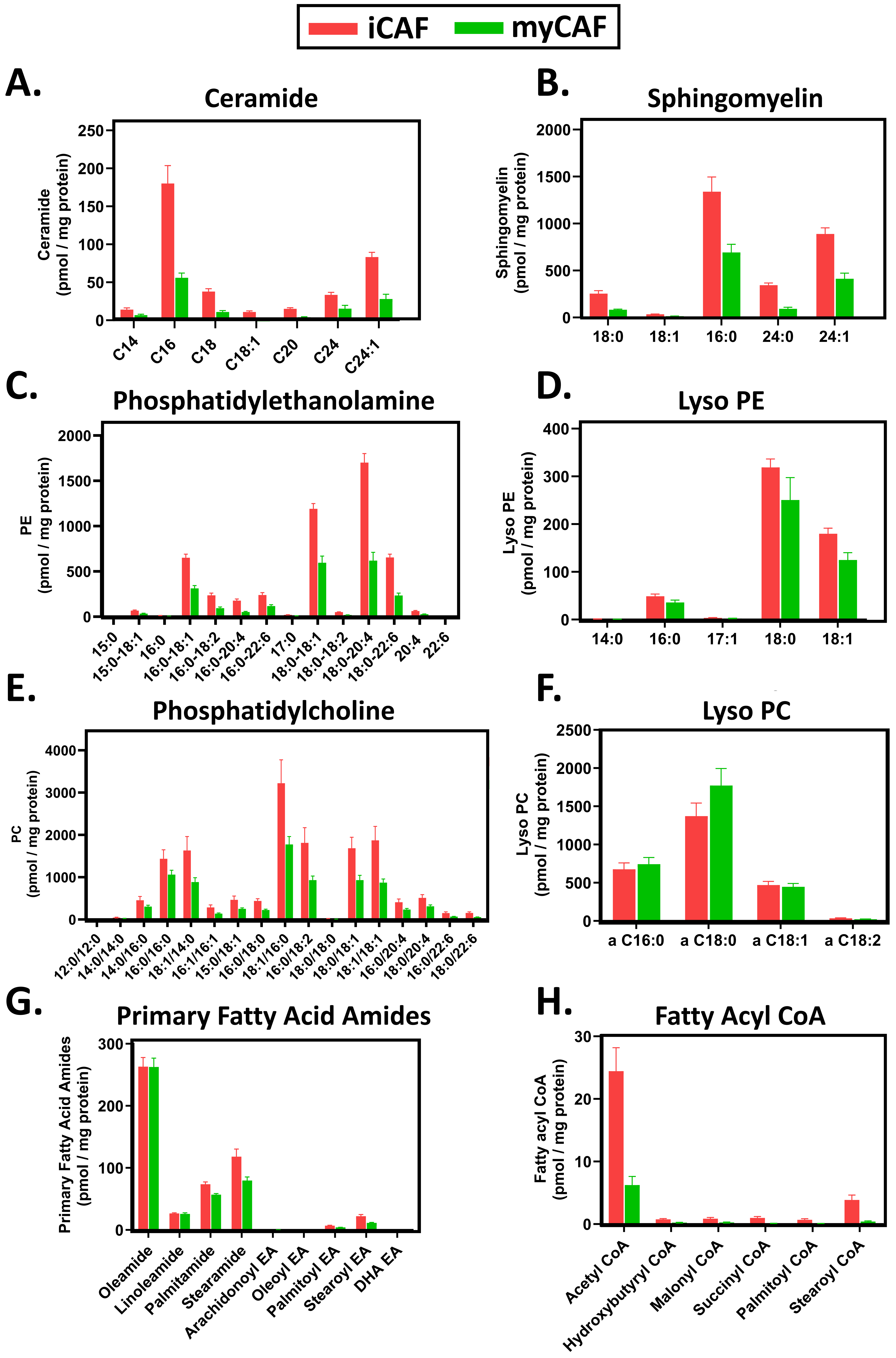

iCAF
myCAF
A.
Ceramide
Ceramide (pmol / mg protein)
0
50
100
150
200
250
C14
C16
C18
C18:1
C20
C24
C24:1
B.
Sphingomyelin
Sphingomyelin (pmol / mg protein)
0
500
1000
1500
2000
18:0
18:1
16:0
24:0
24:1
C.
Phosphatidylethanolamine
PE (pmol / mg protein)
0
500
1000
1500
2000
15:0
15:0-18:1
16:0
16:0-18:1
16:0-18:2
16:0-20:4
16:0-22:6
17:0
18:0-18:1
18:0-18:2
18:0-20:4
18:0-22:6
20:4
22:6
D.
Lyso PE
Lyso PE (pmol / mg protein)
0
100
200
300
400
14:0
16:0
17:1
18:0
18:1
E.
Phosphatidylcholine
PC (pmol / mg protein)
0
1000
2000
3000
4000
12:0/12:0
14:0/14:0
14:0/16:0
16:0/16:0
18:1/14:0
16:1/16:1
15:0/18:1
16:0/18:0
18:1/16:0
16:0/18:2
18:0/18:0
18:0/18:1
18:1/18:1
16:0/20:4
18:0/20:4
16:0/22:6
18:0/22:6
F.
Lyso PC
Lyso PC (pmol / mg protein)
0
500
1000
1500
2000
2500
a C16:0
a C18:0
a C18:1
a C18:2
G.
Primary Fatty Acid Amides
Primary Fatty Acid Amides (pmol / mg protein)
0
100
200
300
Oleamide
Linoleamide
Palmitamide
Stearamide
Arachidonoyl EA
Oleoyl EA
Palmitoyl EA
Stearoyl EA
DHA EA
H.
Fatty Acyl CoA
Fatty acyl CoA (pmol / mg protein)
0
10
20
30
Acetyl CoA
Hydroxybutyryl CoA
Malonyl CoA
Succinyl CoA
Palmitoyl CoA
Stearoyl CoA
