# Supplementary figures and images for: Molecular Profiling of Inflammatory and Myofibroblast Cancer-Associated Fibroblast Subtypes Derived from Human Pancreatic Stellate Cells Using Machine Learning-Based Label-Free Raman Spectroscopy
Source: Biomater Res. 2025 Dec 9;29:0292. doi: 10.34133/bmr.0292 (PMC12686345; doi:10.34133/bmr.0292)

# Box Plot for Raman Spectra Based on VIP Score

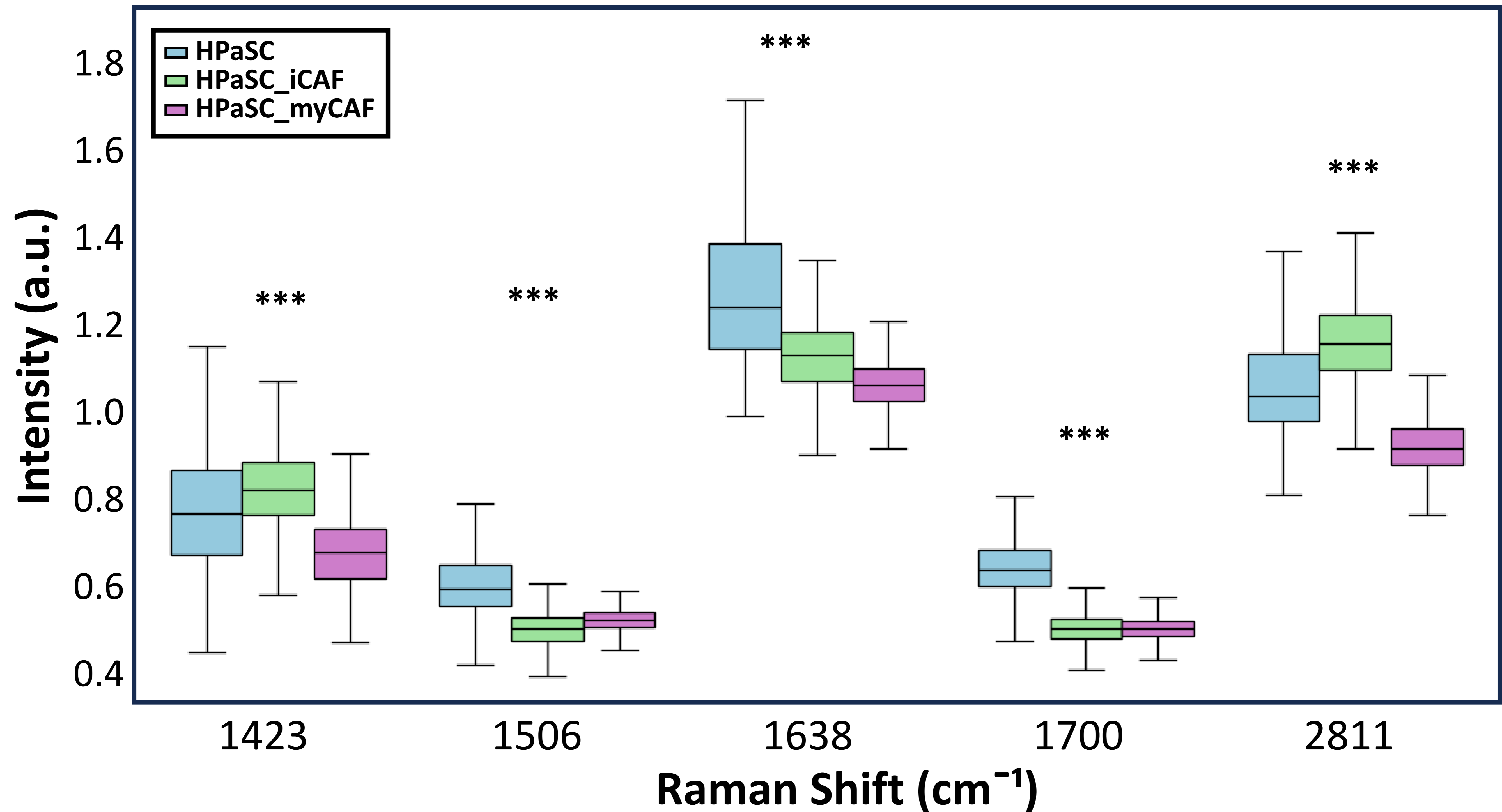

Supplement: Supplementary 1 — Figs. S1 to S4 Table S1 [file bmr.0292.f1.zip › Figure_S1_PDF.pdf]

# Box Plot for Raman Spectra Based on VIP Score

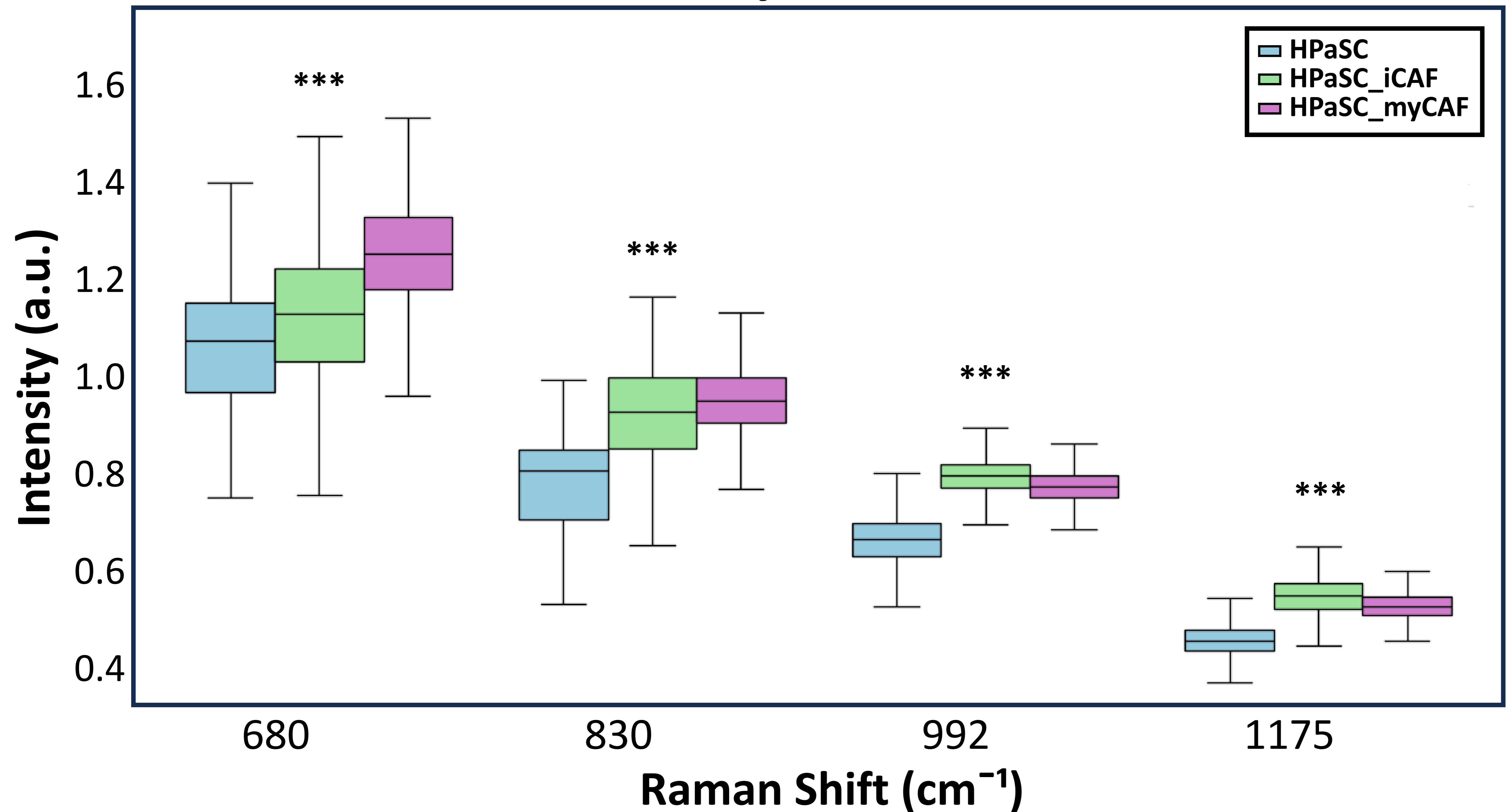

Supplement: Supplementary 1 — Figs. S1 to S4 Table S1 [file bmr.0292.f1.zip › Figure_S2_PDF.pdf]

# Box Plot for Raman Spectra Based on VIP Score

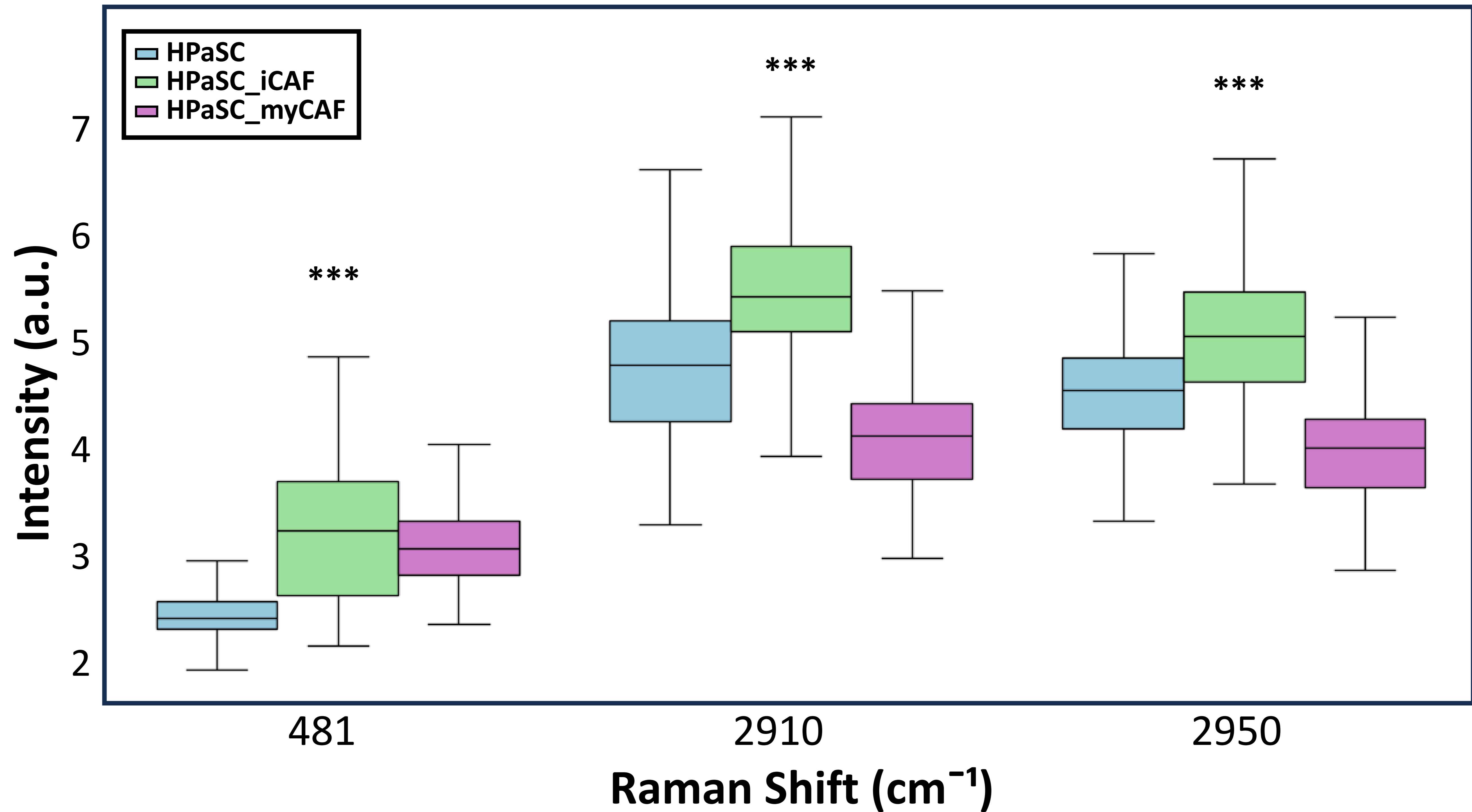

Supplement: Supplementary 1 — Figs. S1 to S4 Table S1 [file bmr.0292.f1.zip › Figure_S3_PDF.pdf]
